# 4D Flow Patterns and Relative Pressure Distribution in a Left Ventricle Model by Shake-the-Box and Proper Orthogonal Decomposition Analysis

**DOI:** 10.1007/s13239-023-00684-0

**Published:** 2023-10-02

**Authors:** Xiaolin Wu, Hicham Saaid, Jason Voorneveld, Tom Claessens, Jos J. M. Westenberg, Nico de Jong, Johan G. Bosch, Saša Kenjereš

**Affiliations:** 1 Department of Chemical Engineering, Faculty of Applied Sciences, Delft University of Technology, Delft, The Netherlands; 2 J. M. Burgerscentrum Research School for Fluid Mechanics, Delft, The Netherlands; 3 Institute Biomedical Technology, Ghent University, Ghent, Belgium; 4 Department of Biomedical Engineering, Thorax Center, Erasmus MC University Medical Center, Rotterdam, The Netherlands; 5 Department of Materials, Textiles and Chemical Engineering, Ghent University, Ghent, Belgium; 6 CardioVascular Imaging Group, Department of Radiology, Leiden University Medical Center, Leiden, The Netherlands

**Keywords:** Left ventricle, Hemodynamics, Pressure, Particle tracking, Shake-the-Box, Proper orthogonal decomposition

## Abstract

**Purpose:**

Intraventricular blood flow dynamics are associated with cardiac function. Accurate, noninvasive, and easy assessments of hemodynamic quantities (such as velocity, vortex, and pressure) could be an important addition to the clinical diagnosis and treatment of heart diseases. However, the complex time-varying flow brings many challenges to the existing noninvasive image-based hemodynamic assessments. The development of reliable techniques and analysis tools is essential for the application of hemodynamic biomarkers in clinical practice.

**Methods:**

In this study, a time-resolved particle tracking method, Shake-the-Box, was applied to reconstruct the flow in a realistic left ventricle (LV) silicone model with biological valves. Based on the obtained velocity, 4D pressure field was calculated using a Poisson equation-based pressure solver. Furthermore, flow analysis by proper orthogonal decomposition (POD) of the 4D velocity field has been performed.

**Results:**

As a result of the Shake-the-Box algorithm, we have extracted: (i) particle positions, (ii) particle tracks, and finally, (iii) 4D velocity fields. From the latter, the temporal evolution of the 3D pressure field during the full cardiac cycle was obtained. The obtained maximal pressure difference extracted along the base-to-apex was about 2.7 mmHg, which is in good agreement with those reported in vivo. The POD analysis results showed a clear picture of different scale of vortices in the pulsatile LV flow, together with their time-varying information and corresponding kinetic energy content. To reconstruct 95% of the kinetic energy of the LV flow, only the first six POD modes would be required, leading to significant data reduction.

**Conclusions:**

This work demonstrated Shake-the-Box is a promising technique to accurately reconstruct the left ventricle flow field in vitro. The good spatial and temporal resolutions of the velocity measurements enabled a 4D reconstruction of the pressure field in the left ventricle. The application of POD analysis showed its potential in reducing the complexity of the high-resolution left ventricle flow measurements. For future work, image analysis, multi-modality flow assessments, and the development of new flow-derived biomarkers can benefit from fast and data-reducing POD analysis.

**Supplementary Information:**

The online version contains supplementary material available at 10.1007/s13239-023-00684-0.

## Introduction

Current research efforts in hemodynamic analysis of blood flow in a left ventricle (LV) are associated with an ever-demanding quest to find proper flow-derived biomarkers that can be used for the early identification of heart failure. Studies have demonstrated that the intraventricular flow patterns, local pressure differences, and flow kinetic energy were closely related to the function, efficiency, and pathologies of the LV [1–3]. For example, patients that underwent mitral valve replacement had a reversed vortical motion in LV during diastole, which resulted in higher flow kinetic energy dissipation compared to the normal subjects [4]. The intraventricular pressure gradient is found to be correlated to the propagation of early diastolic filling in heart failure patients [5]. Based on a similar foundation, many research studies are ongoing to further refine quantitative assessment of the hemodynamic parameters in the LV or to develop new reliable tools for this, which will enable a timely diagnosis of heart diseases in clinical practice.

In literature, two-dimensional (2D) particle imaging velocimetry (PIV) (either in form of a standard planar PIV providing just two velocity components, or in form of stereoscopic PIV providing all three components of the velocity in a characteristic plane) is the most commonly used optical technique for in vitro flow measurements in transparent models of the LV, and downstream of the heart valves [6–9]. However, the vortex-rich flow structures and the wide-ranged flow regimes—from laminar to transitional, and turbulent—within the LV necessitate the use of three-dimensional (3D) high-resolution experimental techniques. Only in recent years, 3D optical techniques, such as tomographic PIV (Tomo-PIV) and 3D particle tracking velocimetry (PTV), are applied in studying the flow downstream of the aortic or mitral valves [10–13]. Despite the fact that Tomo-PIV is a volumetric technique, there are still some technical challenges that affect the obtained velocity field [14] (e.g., ghost particles, limited position accuracy of 0.3 pixel, spatial averaging over interrogation windows resulting in the smoothing of the velocity gradients and small-scale flow structures, etc.).

Compared to PIV, particle tracking velocimetry (PTV) techniques have a higher order of accuracy and are capable of delivering more reliable results in strong-shear flow and near-wall regions [14]. However, there is a major drawback of conventional PTV techniques, which is the limited spatial resolution restricted by low seeding density. Recently, a novel 3D Lagrangian particle tracking (LPT) algorithm ‘Shake-the-Box’ (STB) [14] has been developed. It has overcome the low seeding concentration limitation, allowing the evaluation of significantly denser particle trajectory fields at very low ghost particles occurrence (< 0.004% false particles) and high accuracy (average position error of 0.018 pixel). With data assimilation algorithms or data binning, STB results can provide temporally resolved three-dimensional/three-components (3D/3C) Eulerian velocity fields in a high-resolution manner that is comparable (or even superior) to Tomo-PIV. To date, STB has not yet been applied in studying the flow topology in the LV.

At present, 3D flow diagnostics techniques are becoming more mature and applicable for a wide range of flow phenomena, allowing advanced analysis of the high-resolution velocity field and velocity-derived hemodynamic parameters. Modal analysis techniques such as Proper Orthogonal Decomposition (POD) offer a promising new direction for analyzing blood flow. POD is a data-driven reduced-order modeling tool, which enables breaking down a large high-dimensional dataset into a low-dimensional system where the flow evolution is represented by energy-ordered spatial and temporal modes [15]. The dominant coherent flow structures in complex flows are effectively identified by finite physically interpretable linear modes while the inessential low-energy degrees of freedom are removed. In addition, POD is an entirely data-dependent technique that does not require *a priori* information about the system behavior. Comparative assessments based on the POD modes are computationally quite effective and reliable since they do not require registration nor mesh interpolations to compare datasets with different grids, coordinates, as well as different resolutions. Thanks to these features, POD analysis was widely used in various fluid mechanics applications. In cardiovascular flow, POD has been used in the analysis of the transitional or turbulent flow regimes in the following flows: (i) behind the mechanical aortic valve, (ii) in intracranial aneurysms, and (iii) in abdominal aortic aneurysms [16–18].

The main goals of the present work are: (a) to obtain 4D flow in a realistic LV model with biological valves using Shake-the-Box flow measurement technique; (b) to reconstruct the LV pressure field based on the 4D flow Shake-the-Box measurements; (c) to provide additional spatial-temporal information of the LV flow by applying POD analysis. These results can provide additional insights into the fluid dynamics of heart valves and the left ventricle.

## Methods

### Experiment Set-Up

**Fig. 1:**
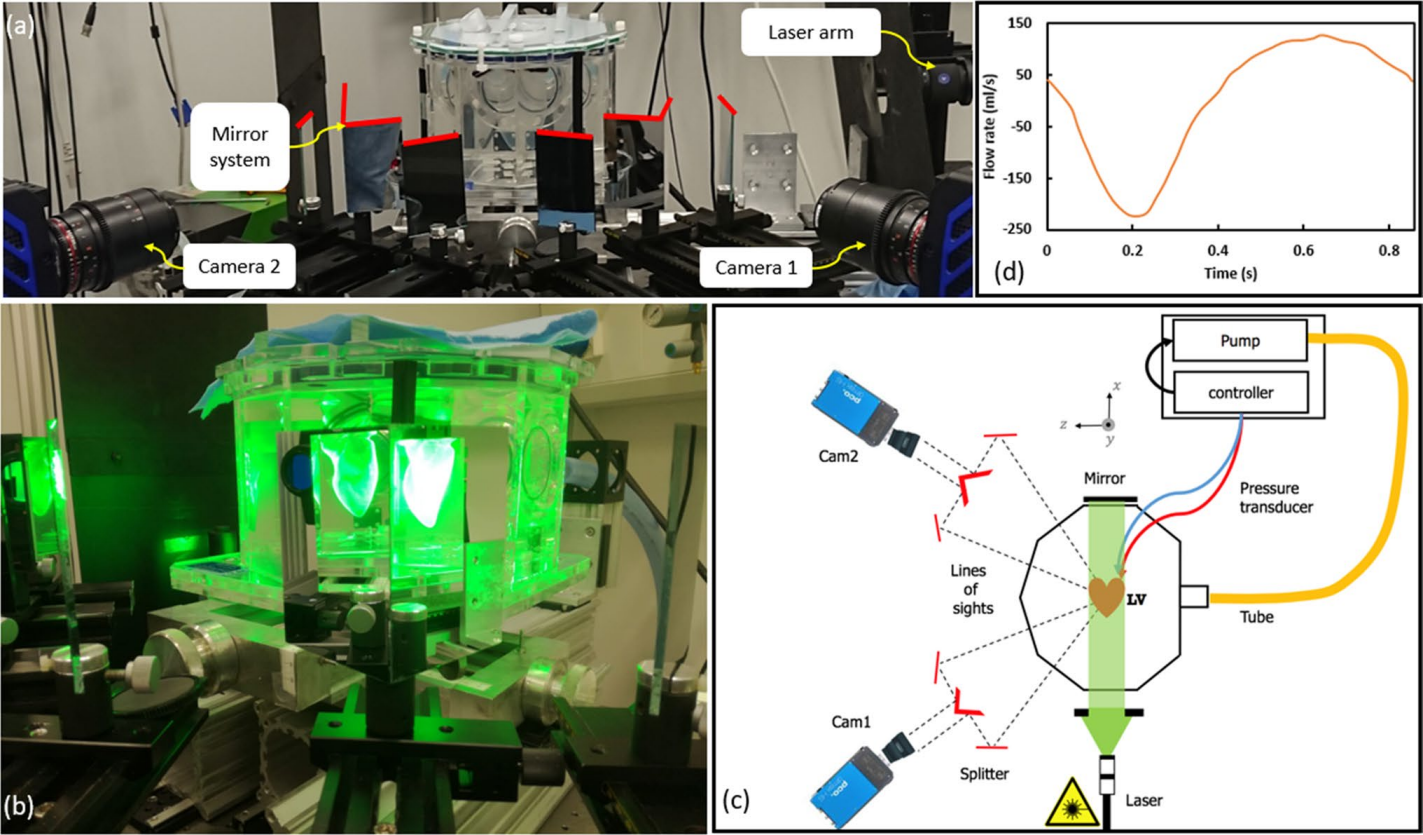
**a** The experimental setup (the front view), consisting of the left ventricle phantom, laser arm, two CMOS cameras, and an image splitter mirror system, **b** An indication of the image splitting, **c** Schematic sketch of the experimental setup (the top-view), **d** The flow waveform imposed at the pump

**Fig. 2:**
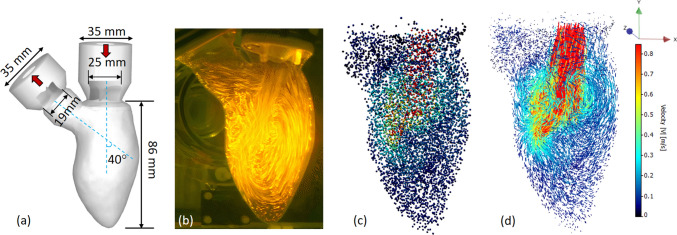
**a** Characteristic dimensions of the left ventricle model. The model contains bio-prosthetic mitral (25 mm, Perimount, Edwards Lifesciences Corp.) and aortic (19 mm, Perimount Magna Ease, Edwards Lifesciences Corp.) valves. The angle between the mitral valve and the aortic valve axises is 40°. **b** A long exposure picture of the particle image of the LV flow. **c** Particle tracers at an instantaneous time step reconstructed from the STB algorithm. **d** Reconstructed particle tracks of 40 consecutive time steps

The realistic LV used in this study has a statistically averaged shape derived from 150 patients’ computed tomography (CT) scans [11, 19, 20]. The optically transparent compliant phantom based on this averaged shape geometry was made of four silicone layers (HT 33 Transparent LT, Zhermack SpA, Rome, Italy). In addition to the LV shape, the phantom also contains a simplified atrium and aortic root extensions. Biological valves (biological Perimount 2900, Edwards Lifesciences, Irvine, USA) were placed at the aortic and mitral positions. Characteristic dimensions of the complete model are summarized in Fig. 2a. The refractive index of the silicone phantom is n = 1.413. To achieve minimal optical distortion, a mixture of water-glycerol was chosen as a blood-mimicking fluid. By varying the water/glycerol concentration, the refractive index was matched to the phantom material. The resulting fluid solution (60% glycerol, 40% water) has a density of *ρ* = 1160 kg/m^3^ and dynamic viscosity of *μ* = 0.0177 Pa s. The fluid in the LV was seeded with Fluorescent Rhodamine-B particles (diameter of 20–50 µm and a density of 1100 kg/m^3^) in a particle image density of 0.04 particles per pixel (ppp). A commercial piston pump (Vivitro Labs Inc., BC, Canada) was used to impose a sinusoidal-like volume change of the external pressure chamber with a cardiac cycle period of 857 ms (70 bpm, systolic duration of 300 ms, stroke volume 50 ml). The dynamics of the LV-shape variation was imposed by this external volume variation (the ventricle volume change must be equivalent to the external volume change) which induces the flow through the valves, mimicking an actively pumping ventricle. The overview of the experimental setup is shown in Fig. 1. For additional details of the experimental configuration, please see [11].

### Data Acquisition and Shake-the-Box Analysis

Two high-speed cameras (Imager Pro HS 4 M, PCO, Kelheim, Germany), each with an image splitter, were applied to acquire particle images from four different views with a frequency of 2 kHz. Image acquisition and processing were performed in DaVis 10.1 (LaVision, Göttingen, Germany). In total 30 cardiac cycles were recorded. The initial calibration was performed using a two-plane target immersed in the working fluid and a third-order polynomial fitting mapping method was applied, resulting in an error of approximately 0.2 pixel (~ 2.4 µm) for all views. This calibration was then improved to less than 0.02 pixel error by conducting volume self-calibration based on preprocessed particle images. Details of the image preprocessing were given in our previous publication [11]. Next, a local determination of the particles' optical transfer function (OTF) [21] was performed to correctly describe the particle shapes in recorded images, which was needed for the STB calculations.

To initialize the STB algorithm, identification of particles was performed in the first four time steps using an iterative particle reconstruction (IPR) method [22]. To find as many potential particle candidates as possible, an intensity threshold of 5 counts and an allowed triangulation error of 1 voxel were specified. After initialization, an iterative process was applied for the subsequent time steps in adding particles and refining their positions by matching the images (i.e., ‘shaking’ procedure). For each time instant, a guess of the particles' next position was made by extrapolating particle projections from the previous time steps. The predicted distribution of particles was refined by shaking particles by 0.1 voxel in the 3D domain. Simultaneously, residual images were obtained by subtracting the corrected particles distribution from the initial images. Then, a triangulation was conducted on the residual images to identify new particles. Both the tracked and new particles were shaken again in a total of eight iterations to increase the accuracy. New particles were added only if they were found in at least four consecutive time steps. The final convergent solution contained approximately 7000 particle tracks in the measured volume (62 × 100 × 57 mm). To obtain the final time-resolved 3D velocity field and derived variables (e.g., vorticity, vortical structures, or pressure), the particle tracks were first fitted with quadratic B-splines with a typical length of 3 time steps to retrieve velocity and acceleration from the discrete particle-based information. After this, the velocity vectors were interpolated onto the Eulerian grid using binning with the Gaussian weighting. The data Binning is similar to the interrogation window in the Tomo-PIV. In the vector Binning process, we set an initial sub-volume of 72 voxels and an overlap of 87.5% for iterations, resulting in a final resolution of 9 voxels (0.735 mm) in each dimension. A convergence analysis was conducted by comparing the phase averaged data obtained from 10, 20, and 30 cardiac cycles. We obtained velocity vector differences smaller than 2% among these datasets. Hence, the averaged velocity field data of 10 cycles is sufficient to obtain a reliable POD analysis and pressure calculations.

### Instantaneous Pressure Field

To obtain the relative pressure field from the STB calculated velocity vectors, we apply the 4D Poisson solver of DaVis 10.1. We provide a short mathematical rationale of the pressure extraction based on the Navier-Stokes equations for incompressible fluids [23]:

1 $$\begin{array}{c}\nabla p=-\rho \frac{D{\varvec{u}}}{Dt}+\mu {\nabla }^{2}u\end{array}$$

where *p*, ***u***, *t*, *ρ,* and *μ* represent the pressure, velocity, density, and dynamic viscosity of the fluid, respectively, and $\frac{D{\varvec{u}}}{Dt}$ is the velocity material derivative. The latter can be expressed in the Lagrangian framework as a local velocity difference of a particle at two points along its pathline:

2 $$\begin{array}{c}\frac{D{\varvec{u}}}{Dt}=\frac{d{{\varvec{u}}}_{p}\left(t\right)}{dt}=\frac{d{\varvec{u}}\left({{\varvec{x}}}_{p}\left(t\right),t\right)}{dt}\end{array}$$

where ***x***_*p*_(*t*) and ***u***_*p*_(*t*) are the position and the velocity of a selected fluid particle, respectively. The particle positions ***x***_*p*_ are determined from the STB reconstructed particle trajectories, and the material derivative is calculated from the second-order least-square fitting scheme. Once the material derivative is determined, the pressure gradient of the entire measurement domain can be obtained from Eq. (1). The pressure field is then evaluated by solving a Poisson equation. By taking the divergence of Eq. (1), one can obtain the Poisson pressure equation (PPE):

3 $$\begin{array}{c}{\nabla }^{2}p=\nabla \cdot b\end{array}$$

where ***b*** denotes contributions from the right-handed side terms of Eq. (1). In Davis, the 4D pressure solver also includes the time derivative term of pressure via estimation of the convective velocity of the flow structure:

4 $$\begin{array}{c}{\nabla }^{{\prime}2}p=\nabla \cdot b+{\upxi \left.\frac{{\partial }^{2}p}{{\partial t}^{2}}\right|}_{c}\end{array}$$

where ${\nabla }^{{\prime}2}$ is a modified operator, ξ is the weighting factor between the temporal and spatial derivatives, and the time derivative of pressure is calculated as:

5 $$\begin{array}{c}{\left.\frac{\partial p}{\partial t}\right|}_{c}\approx \frac{1}{\Delta t}{\int }_{x+{{\varvec{u}}}_{c}\Delta t}^{x}\nabla pd{\varvec{x}}\end{array}$$

where ***u***_*c*_ is the convective velocity. If setting ξ = 0, then the convective velocity is not calculated and the pressure field is evaluated separately for each instantaneous velocity field. In the present study, we adopt ξ = 1, resulting in the estimation of the convective velocity ***u***_*c*_ by applying the least-squares method of [24]. Moreover, under the incompressible fluid assumption, a divergence-free filter is applied to the calculated velocity field before it is used for the pressure calculation. This leads to efficient suppression of non-physical pressure fluctuations in time, resulting in high temporal accuracy. An additional advantage of the inclusion of the time derivative of the pressure in the PPE equation is that a reference pressure only needs to be applied once at the very first time step. In the present study, we prescribed Dirichlet boundary condition with an average zero value of the measured domain at the first time step. Whereas Neumann condition, which makes use of the calculated pressure gradient from Eq. 1, is imposed at all boundary voxels at each time step. Consequently, the calculated field represents the relative pressure. Additional numerical details of the 4D pressure solver are given in [25, 26].

### 3D POD Analysis

Next, we present the mathematical rationale of the snapshot POD analysis that we applied to the STB measured instantaneous velocity field [27, 28]. Each instantaneous STB velocity measurement is a snapshot of the flow. In the snapshot POD method, the velocity field is decomposed into a linear combination of spatially orthonormal modes and their corresponding coefficients:

6 $$\begin{array}{*{20}c} {u\left( {x,t} \right) \approx \sum\limits_{{k = 1}}^{N} {a_{k} } \left( t \right) \cdot \phi _{k} \left( x \right)} \\ \end{array}$$

where *x* is the location, *t* is the time, *N* denotes the total number of snapshots (*k* is the snapshot index), *u*(*x*, *t*) is the measured velocity, ${\phi }_{k}\left(x\right)$ are the spatial orthonormal modes (i.e. eigenfunctions representing the spatial behavior of the flow), and *a*_*k*_(*t*) are the time coefficients (representing the flow changes over time). The eigenfunctions are obtained by solving the eigenvalue problem. In the content of the present work, the resulting eigenvalues will be referred to as the kinetic energy contributions of corresponding POD modes [28, 29]. These eigenvalues then can be ordered in a decreasing order such that the first few POD modes are containing the largest amount of kinetic energy. Consequently, dominant coherent flow structures associated with the first few POD modes can be easily identified. In the present study, the full three-dimensional POD analysis is performed over 343 snapshots (5× temporal subsampling of STB data) covering the cardiac cycle. Note that the shape of the left ventricle model changes over time. This moving boundary condition results the grid and the number of vectors differs from each snapshot. To handle this condition, the mesh at end-systole was selected as reference frame. The velocity data of other time steps were interpolated by polynomial functions onto this reference mesh. The POD analysis is then performed on the interpolated data.

## Results and Discussion

### General Intraventricular Flow Features

**Fig. 3:**
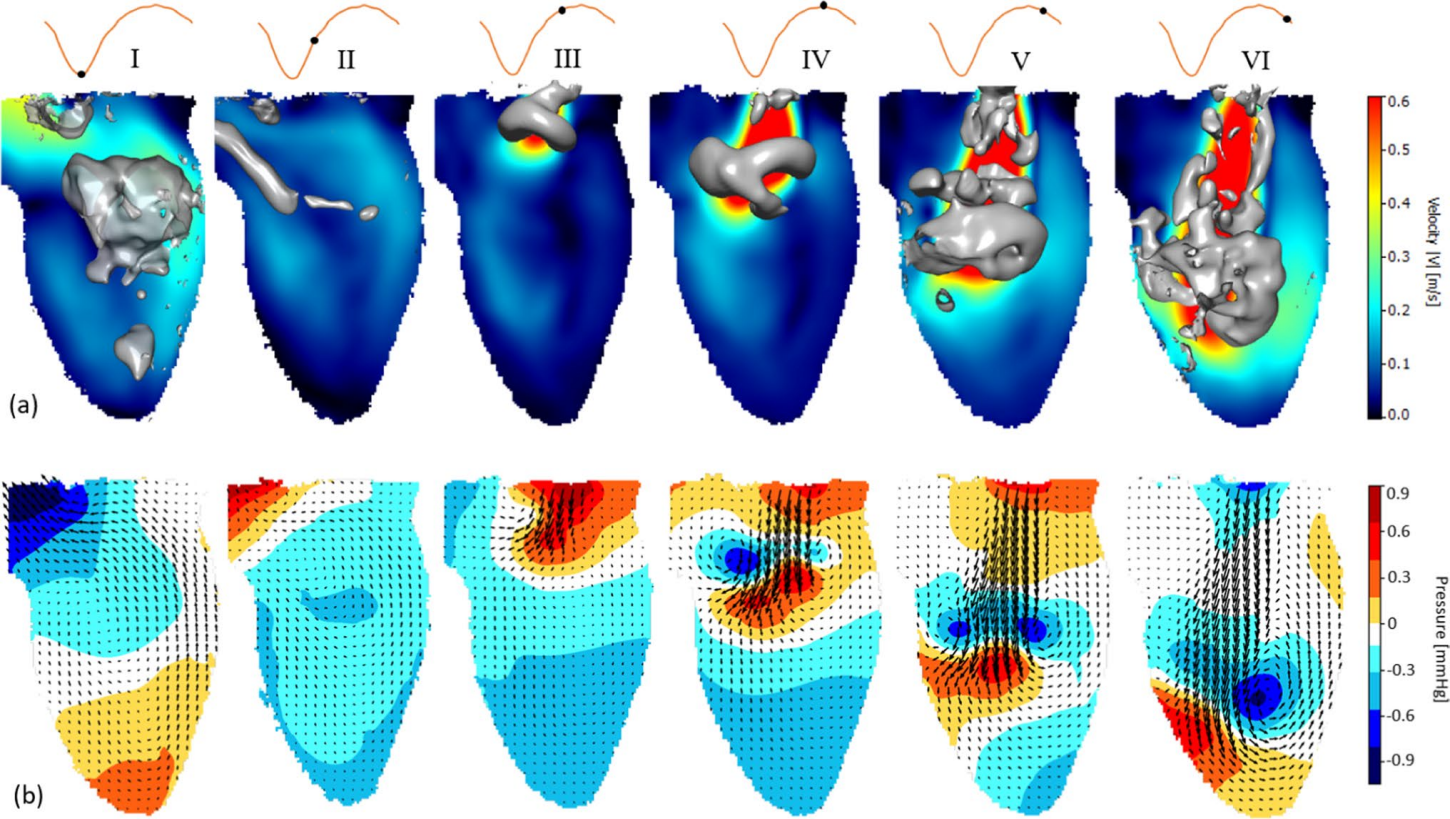
**a** Velocity contours at the center 2D z-plane and the 3D vortical structures identified by the lambda-2 criterion are visualized as iso-surface (λ_2_ = − 150 1/s^2^). **b** Instantaneous Pressure contour at the center 2D z-plane. Roman numerals indicate the time steps (I = 0.265 s, II = 0.4 s, III = 0.645 s, IV = 0.7 s, V = 0.765 s, VI = 0.85 s)

The generated flow structures in the LV are visualized by means of a long exposure recording of the fluorescent particles during Tomo-PIV experiments [11], shown in Fig. 2b. Corresponding instantaneous locations of the particles extracted from the Shake-the-Box are shown in Fig. 2c. The reconstructed particle tracks from 40 consecutive time steps during the filling phase are shown in Fig. 2d. Approximately 7000 particles are identified and colored by the velocity magnitude. It can be seen that the biological valve generates a strong trans-mitral jet accompanied by a pair of counter-rotating vortices, Fig. 2d. This particular feature has been regarded as an important criterion in valve design. Many studies have addressed that optimal vortex formation plays an important role in preserving the momentum and kinetic energy of the intraventricular flow [3, 30, 31]. In Fig. 3a, we tracked the diastolic vortex propagation by presenting vortex structures at six characteristic time instants of the cardiac cycle. Here, we superimposed the contours of the velocity magnitude in the central vertical plane with 3D vortex structures identified as iso-surfaces of the λ_2_ = − 150 1/s^2^ (colored in gray) (where λ_2_ is the second eigenvalue of the symmetric tensor (*S*2* + Ω*2) where *S* and *Ω* are symmetric and antisymmetric parts of the velocity gradient tensor [32]). The roll-up of the shear layer, generated by the transmitral flow jet, results in a ring-shaped vortex. It starts to pinch-off from the mitral annulus at time step III, and then propagates towards the center of the ventricle at later time instants IV and V. At time instant VI, the central jet reaches the side wall of the ventricle and the dominant vortex ring structure starts to break down. The evolution of this vortical structure is in agreement with similar in vivo and in silico studies previously reported in literature [3, 30, 31]. We perform a detailed comparison of the STB measured velocity field with the previous Tomo-PIV (see the Supplementary Material). Overall good agreement between techniques is obtained with some distinct advantages of the STB in a better representation of the velocity vectors in the near wall region and, consequently, in a more accurate capturing of the LV wall morphology changes during the cardiac cycle (see animations in Supplementary Material). This can be beneficial for the validation of numerical simulations intended to mimic exact experimental conditions, similar to Refs. [11, 33].

### 4D Relative Pressure Field

**Fig. 4:**
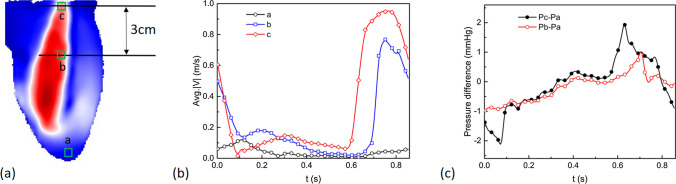
Assessments of temporal velocity profiles (**b**) at different positions **a**–**c** shown in (**a**). Pressure differences (**c**) between the apex location **a** and location near the left ventricle mitral annulus **b** and the base **c**. The pressure and velocity were averaged in a 2 × 2 mm box (green) located at the center 2D z-plane

In Fig. 3b, we show the relative pressure contours in the central vertical plane at various time instants of the cardiac cycle. 2D projections of the velocity vectors (x- and y-components) are superimposed on the pressure contour to indicate the flow. To further study the temporal variation of the intraventricular pressure, the pressure differences between two representative locations and the apex of the LV are plotted over time in Fig. 4c. The locations are indicated in Fig. 4a: (a) the apex, (b) 3 cm below location c, and (c) the center of the mitral annulus. The velocity temporal profiles of these locations are also plotted in Fig. 4b. The diastolic and systolic flow can be recognized from the velocity profile at the LV base (location c).

The results show that the pressure distribution along the (longitudinal) base-apex axis reverses at the onset and the termination of the LV filling and ejecting, respectively (Fig. 3b). At the onset of early diastole (i.e., time-instant III), high pressure is observed near the mitral valve and is gradually decreasing towards the LV apex, driving the blood entering the LV rapidly. Despite the rapid filling, the pressure in the proximity of the apex is continuously decreasing (time-instant IV), which can be observed as well in a successful LV suction in vivo. After the completion of the ventricular filling, a pressure increase takes place near the apex, and the pressure difference between the base and the apex starts to decline (time-step V), resulting in the blood flow decelerating and redirecting. Eventually, a reversal of the initial pressure distribution is observed at the late diastole (time-step VI): low pressure at the base while high pressure at the apex. Notably, during diastole, the intraventricular pressure does not increase or decrease monotonically across the long axis of the LV. Instead, localized low-pressure regions are observed where the aforementioned vortex ring presents. During systole, the pressure gradient inside the LV rises again with the minimal local pressure located below the aortic valve and the maximum pressure at the apex, compelling blood flow to the aortic valve (time-instant I). At the end of the systole (time-instant II), the pressure gradient reverses again, with the high- and low-pressure regions located in the proximities of the aortic valve and apex, respectively. This reversion pattern of intraventricular pressure can be also observed from the pressure differences between the apex and the base over time, Fig. 4c. This pressure change pattern in early filling and late ejection contributes to the closure of the aortic and mitral valves [34]. On the other hand, there are also some observed differences compared to the Non-invasive MRI-based relative pressure studies of [35, 36]. In comparison to these studies, the present study exhibits a smaller amplitude of the peak of the temporal evolution of the pressure difference between the apex and basal (mitral valve) locations, and also there is a lack of the second peak. These differences arise due to the simplified sinusoidal flow waveform imposed in our study, which fails to capture the strong acceleration of the E-wave and the presence of A-wave described in vivo. In addition, it’s worth addressing that the spatiotemporal resolution and the choice of sampling points or planes to define the basal and apical locations can affect the accuracy of the pressure assessment, as discussed by [36]. However, it should be noted that the spatial resolution (0.735 × 0.735 × 0.735 mm^3^) and temporal resolution (2 kHz) of current study are considerably fine resolutions.

The overall reconstructed pressure magnitude in the LV model is in a similar range to our CFD result shown in [37], as well as previous studies based on pressure transducers [38], catheters [39], and MRI measurement [40]. The maximum relative pressure range in the LV is found to be about 2.7 mmHg, which is in good agreement with values reported in MRI studies [36, 41–43]. Nevertheless, it is important to acknowledge the impact of the aforementioned limitation, specifically the use of a simplified sinusoidal flow waveform. Despite this limitation, there are some advantages of the STB-based intraventricular relative pressure: (i) it is a non-invasive technique, (ii) it provides time-resolved 3D pressure distributions, instead of the 1-D point-like catheter measurements, (iii) relatively high spatiotemporal resolution, which can be utilized for further calibration, validation and improvement of the clinically available echocardiography [44] and MRI techniques [45].

### POD Analysis

#### POD Modes and Coefficients

**Fig. 5:**
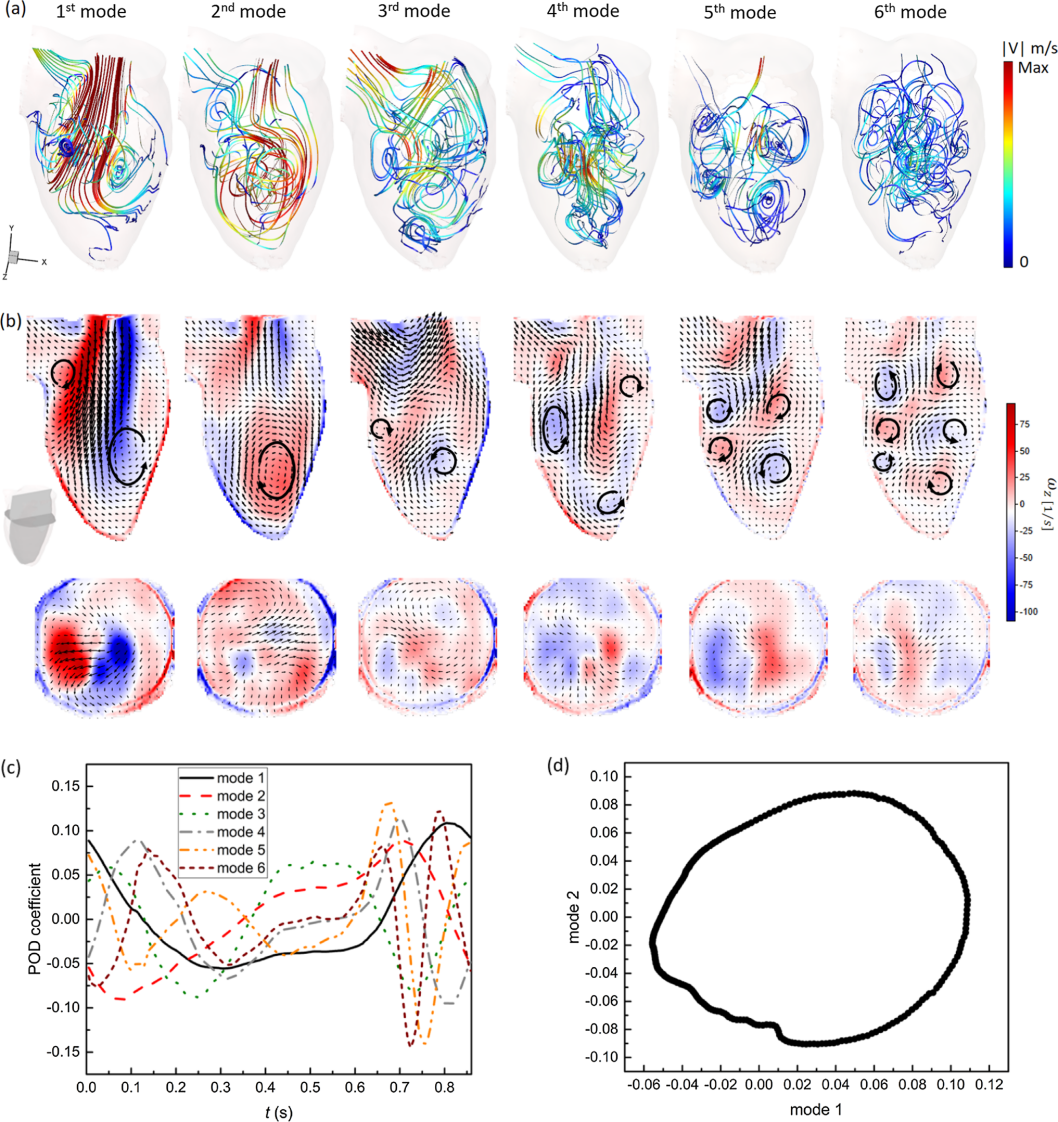
Snapshot POD analysis of the velocity field of the full cardiac cycle: **a** 3D Streamlines for the first six POD spatial modes, colored by velocity magnitude. The maximum of |V| is 0.8 m/s for the first three modes, and is 0.34 m/s for the 4th to 6th modes, **b** Vector fields of the first six POD spatial modes at the center z-plane colored by z-vorticity magnitude, **c** The corresponding time coefficient of the first six POD modes, **d** Phase portrait of the first two POD modes

**Fig. 6:**
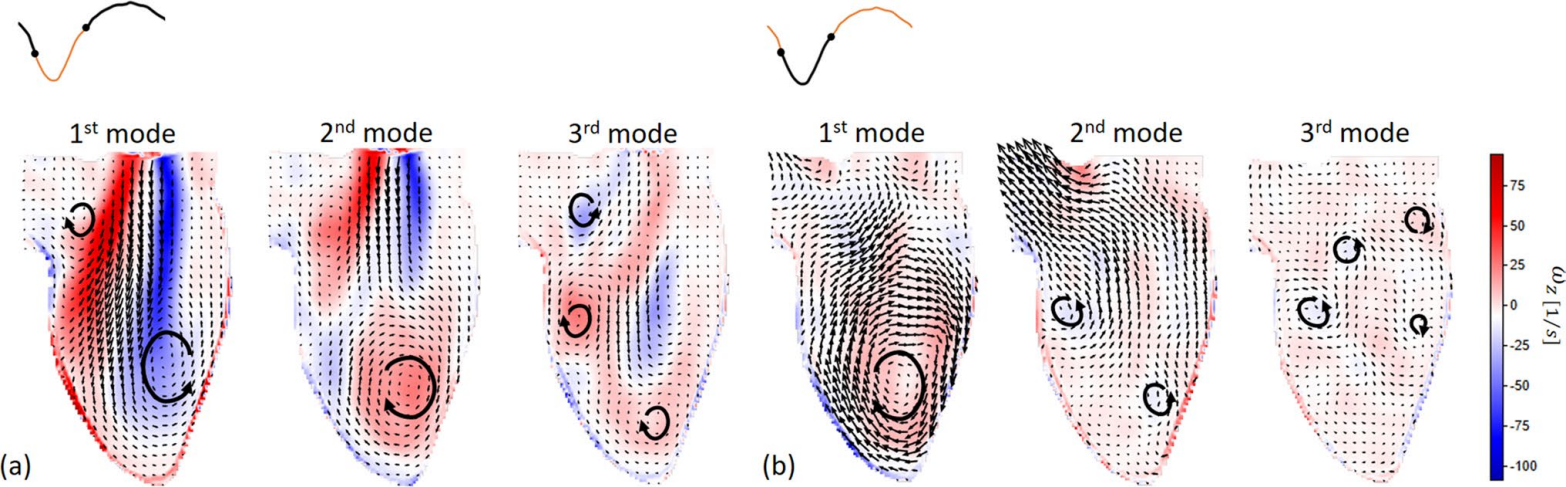
Vector fields of the first three spatial modes at the center z-plane colored by z-vorticity magnitude from **a** POD analysis of the diastolic phase and **b** POD analysis of the systolic phase

Next, we performed the 3D POD analysis using in total 343 snapshots of the STB-based velocity field over the entire cardiac cycle. The resulting flow morphologies visualized by calculated pathlines (colored by the velocity magnitude) for the first six spatial POD modes are shown in Fig. 5a. The corresponding contours of the out-of-plane vorticity component (in the z-coordinate direction) superimposed with the 3D velocity vectors in the central vertical plane are given in Fig. 5b-top. We also indicate the approximate size and direction of rotation of coherent flow structures with the black arrowed lines. Similarly, the contours of the z-vorticity in the selected horizontal plane for identical POD modes are shown in Fig. 5b-bottom. We show this plane also to illustrate the importance of performing a fully 3D POD analysis since by focusing solely on the central vertical plane (i.e., 2D data), many important 3D flow features will not be captured (e.g., the eccentricity of the central trans-mitral jet in the horizontal plane). Recall that the POD modes are ordered by flow kinetic energy dominance such that the first few modes should identify the most distinct flow features. It can be seen that the 1st spatial POD mode captures the central jet surrounded by a pair of counter-rotating vortices. This pattern is a well-known feature of the intraventricular flow during early diastole, as presented in [11, 30, 31]. The 2nd spatial POD mode is characterized by a clockwise coherent flow structure located between the middle of the LV and its apex. This flow structure is associated with vortices that appear during late diastole and remain until early systole. While the first pair of POD modes displayed the most prominent features of LV flow, small-scale flow structures are revealed in the higher spatial POD modes, Fig. 5a and b. The number of these small vortical structures significantly increases for the higher POD modes, but they are characterized by weaker velocity and vorticity. To elucidate further on the above-discussed vortex near the LV apex, we also perform additional POD analysis of the separate systole and diastole phases of the cardiac cycle, respectively, Fig. 6. A similar vortex can be seen in the 2nd POD mode of the diastole (containing 27.9% of total kinetic energy) and the 1st POD mode of the systole phase (containing 63.3% of the kinetic energy). The existence of this vortex was associated with a minimization of the momentum loss during the ejection phase by redirecting the flow already to the aortic root before the LV ejects [4, 46–49]. However, some studies suggested that instead of leading to energy reduction for systolic ejection, this vortical motion is crucial in washing out the blood from the apical region and therefore preventing possible thrombus formation [50–52]. The results presented here indicate that the formation of this vortex is closely correlated with the LV contraction function due to its relatively high energy content (27.9% of diastolic flow, 63.3% of the systolic flow).

In Fig. 5c, the time coefficients of the six POD modes are plotted as a function of the cardiac cycle. It can be seen that the temporal evolutions of the first and second modes are similar and both resemble the shape of the flow waveform imposed at the mitral orifice. A phase-shift of π/4 can be observed between the first two modes. For the higher POD modes, coefficients exhibit a more irregular behavior with various frequencies. However, similar to the first two modes, the coefficients of the second and third pair of modes evolve analogously in a pair, but with a phase-shift. It is noted that this pairwise characteristic was reported in well-defined periodic flows, such as flow over a cylinder [53, 54] (vortex shedding), or flow in carotid artery bifurcation [55]. In periodic flows, the first pair of modes are often found to be the orthogonal components of the basic harmonics and is usually associated with the traveling of a wave. The high temporal correlation of the first two modes can be verified from a phase portrait plotting one time coefficient as a function of another. As Fig. 5d shows, the time coefficients *a*_*1*_ and *a*_*2*_, are strongly correlated, forming an ellipse-like trajectory. The observed deviations from an ideal elliptic trajectory could be an indication of cycle-to-cycle variations, induced by small-scale fluctuations or turbulence.

#### Energy Contribution of POD Modes

**Fig. 7:**
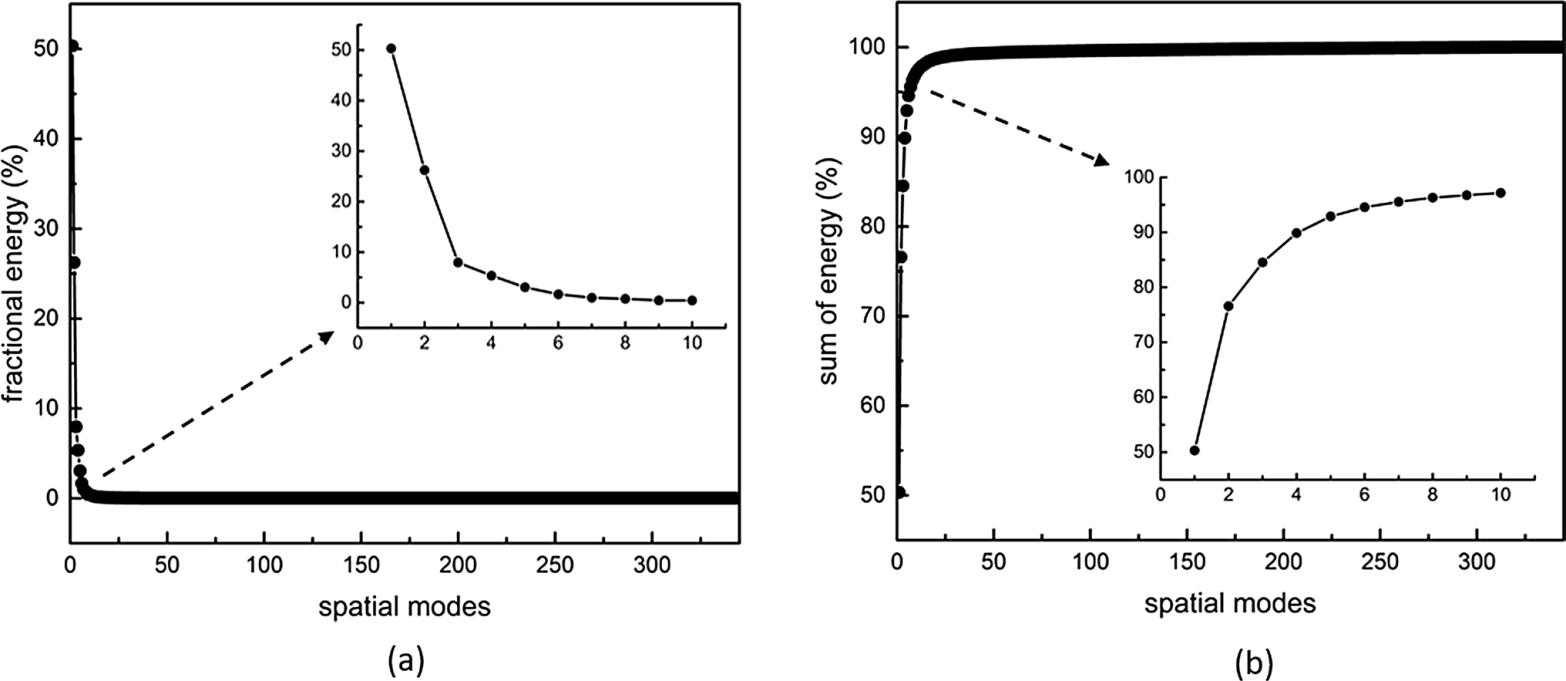
**a** Fraction of total kinetic energy and **b** accumulated fraction of total kinetic energy as a function of the number of modes

The fraction and accumulated fraction of the total kinetic energy of the flow as a function of the POD modes number are shown in Fig. 7a and b, respectively. It can be seen that a sum of the first two POD modes contribute to 76.5% of the total kinetic energy (with 50.3% for the 1st POD and 26.2% for the 2nd POD mode). This confirms the dominance of the first pair of the POD modes (i.e. a superposition of the central mitral jet and vortex near the apex will provide the most salient flow features quite accurately, see Fig. 5a and b). Although the first two eigenvalues are much bigger than the rest, the next four eigenvalues are still bigger than 1%. In total, the second and third pair of POD modes account for 18.5% of the kinetic energy. This suggests that to reconstructed the LV flow with 95% of the kinetic energy content, only six POD modes are needed. Compared to 343 snapshots, a significant data reduction is achieved. It should be kept in mind that the energy contribution of each mode could be case-related. For example, it was reported in experimental studies of a flow in ascending aorta with a normal and dysfunctional bileaflet mechanical aortic valve, the corresponding energetic contributions of the 1st POD mode is approximately 74% and 60%, respectively [16]. Moreover, it was also reported that the normally functioning valve case also required the lowest number of POD modes to reconstruct the flow regardless of the percentage of the kinetic energy to be captured. A similar strategy can be also adopted for the flow in LV where various patient-specific cases and their comparison with the healthy control group can lead to establishing the specific thresholds of the POD modes contributions. An example is the study by [56], which provided 2D POD and DMD analysis for both healthy LV flow and for cases subjected to various degrees of aortic regurgitation. Future studies are needed to extend the database from 2D to 3D POD models. In addition, POD studies based on in vivo measurements are required to establish appropriate clinical thresholds.

### Limitations

Next, we will address some limitations of the performed experimental study. Firstly, the imposed flow and pressure have a simple sinusoidal variation, which does not represent the diastasis and late filling (A-wave) phases of the LV in vivo. Secondly, the blood-mimicking fluid has a 4 times higher viscosity than blood. This could have impact on the flow structure dynamics and corresponding vortex ring formation, pressure, and viscous dissipation rate. In addition, the impact of spatial resolution on the POD analysis has not been investigated. Future study is necessary to quantify its role when applying POD to MRI measurements, considering that MRI technique often has lower spatial resolution than this study. The effect of temporal resolution on POD was studied and the results can be found in the supplementary material. Only 1.0% to 1.6% differences in kinetic energy content were found in the first four modes.

## Conclusions

In this work, we applied Shake-the-Box, an advanced state-of-the-art 3D Lagrangian particle tracking method, to study the flow in a compliant LV model with biological valves. New features such as particle tracks were extracted and a high spatiotemporal resolution of 4D velocity field was resolved. The 4D relative pressure was calculated using a Poisson equation-based solver. Detailed spatial distribution from the base to the apex and temporal evolution of relative pressure over the cardiac cycle were presented. In general, the obtained flow features and relative pressure are in good agreement with those in -vivo reports. In addition, we applied POD analysis to decompose the complex velocity data and extract key information about the flow dynamics in LV flows successfully. Our results showed that the first six POD modes captured 91% of the kinetic energy and provided a clear picture of different scales of vortices formed in the pulsatile LV flow, demonstrating that POD can be an alternative approach to efficiently visualize and analyze the various scale flow structures and their temporal behaviors in the LV.

In summary, this work demonstrated that Shake-the-Box and POD analysis are promising tools for the accurate and efficient investigation of highly three-dimensional and time-varying flows. The obtained new features such as particle tracks and 4D relative pressure results can be used to validate in vivo or in silico LV flows. Clinical image analysis and multi-modality comparison research could benefit from fast, complexity-reduced, and data-reduced POD analysis. Moreover, POD-based metrics can be developed as potential new biomarkers for cardiac function in future work.

### Supplementary Information

Below is the link to the electronic supplementary material.

Supplementary file1 (PDF 623 KB)

Supplementary file2 (MP4 11613 KB)

Supplementary file3 (MP4 44725 KB)

Supplementary file4 (MP4 14972 KB)
