# Supplementary material for: 4D Flow Patterns and Relative Pressure Distribution in a Left Ventricle Model by Shake-the-Box and Proper Orthogonal Decomposition Analysis
Source: Cardiovasc Eng Technol. 2023 Oct 2;14(6):743–54. doi: 10.1007/s13239-023-00684-0 (PMC10739257; doi:10.1007/s13239-023-00684-0)
Supplement: Supplementary file 1 — Supplementary file1 (PDF 623 KB) [file 13239_2023_684_MOESM1_ESM.pdf]

4D Flow Patterns and Relative Pressure Distribution in a Left Ventricle Model by Shake-the-Box and Proper Orthogonal Decomposition Analysis

Xiaolin Wu[1,2,*], Hicham Saaid[3], Jason Voorneveld[4], Tom Claessens[5], Jos J. M. Westenberg[6], Nico de Jong[4], Johan G. Bosch[4], Saša Kenjereš[1,2]

[1]Department of Chemical Engineering, Faculty of Applied Sciences, Delft University of Technology, Delft, The Netherlands

[2]J. M. Burgerscentrum Research School for Fluid Mechanics, Delft, The Netherlands

[3]Institute Biomedical Technology, Ghent University, Ghent, Belgium

[4]Department of Biomedical Engineering, Thorax Center, Erasmus MC University Medical Center, Rotterdam, The Netherlands

[5]Department of Materials, Textiles and Chemical Engineering, Ghent University, Ghent, Belgium

[6]CardioVascular Imaging Group, Department of Radiology, Leiden University Medical Center, Leiden, The Netherlands

*Correspondence: X.Wu-5@tudelft.nl

Supplementary materials

1. Velocity profile comparison with Tomo-PIV results
2. Effect of temporal resolutions on POD
3. Animations of particle tracks, velocity and vorticity

Tomo VS STB velocity results comparison

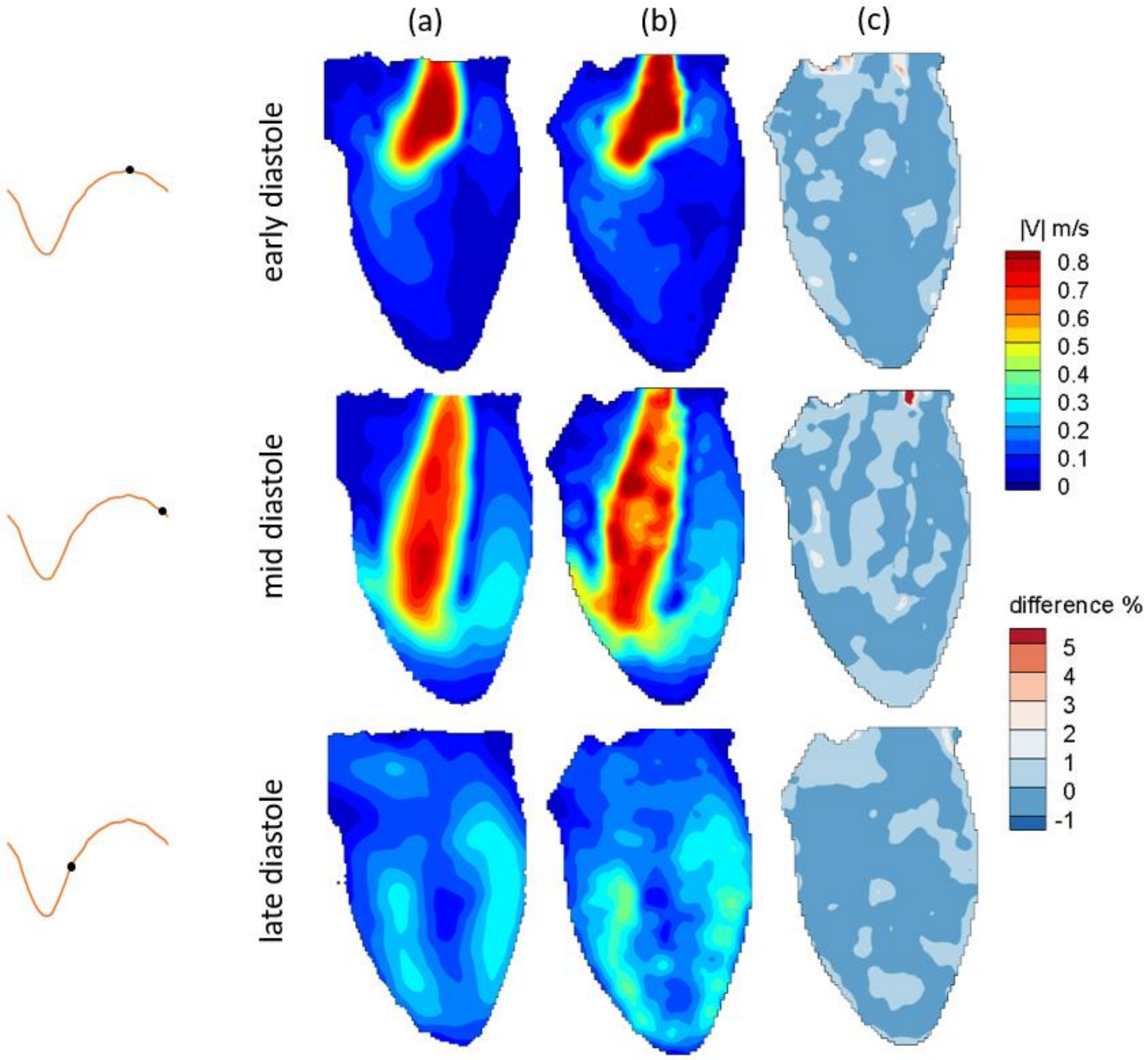

Figure 1. (a) STB results, (b) Tomo-PIV results, (c) percentage of difference between Tomo-PIV and STB

Effect of Temporal resolutions

To examine the sensitivity of the POD results, a statistical convergence test was conducted using data subsets size of 21, 43, 86, 171, 286, 343, 428, and 571 snapshots, corresponding to frame rates of 25, 50, 100, 200, 330, 400, 500, and 660 Hz, respectively. The fractional energy of the first four eigenvalues are plotted as a function of the data size (i.e. number of snapshots) in Fig.2. It reveals that starting from 171 to 571 snapshots (from 200 to 660 Hz frame rate), the magnitude of the fractional energy contributed by the first four eigenvalues does not change significantly, indicating that the present data size (343 snapshots) is sufficiently large for providing statistically converged POD results. Investigating the impact of temporal solution on POD results is essential step, especially considering applying POD to *in vivo* data analysis. *In vivo* blood flow measurement techniques, such PC-MRI and echo-PIV, have limited temporal and spatial resolutions comparing to time-resolved PIV system. Our convergence test result shows that a imaging frame rate of 25 to 100 Hz (temporal

resolution of 40 to 10 ms), matching the temporal resolution range of PC-MRI measurements, led to a variation of 1.5% , 1.6 %, 1.0%, and 1.5% in the kinetic energy content of the first four mode, respectively. If one considers the variations are acceptable, POD could be applied to clinical flow kinetic energy analysis. Together with the low-order reconstruction, flow structures and their temporal behaviors, such as the vortex formation, evolution and convection, can be analyzed in greater detail without requiring high imaging rate data.

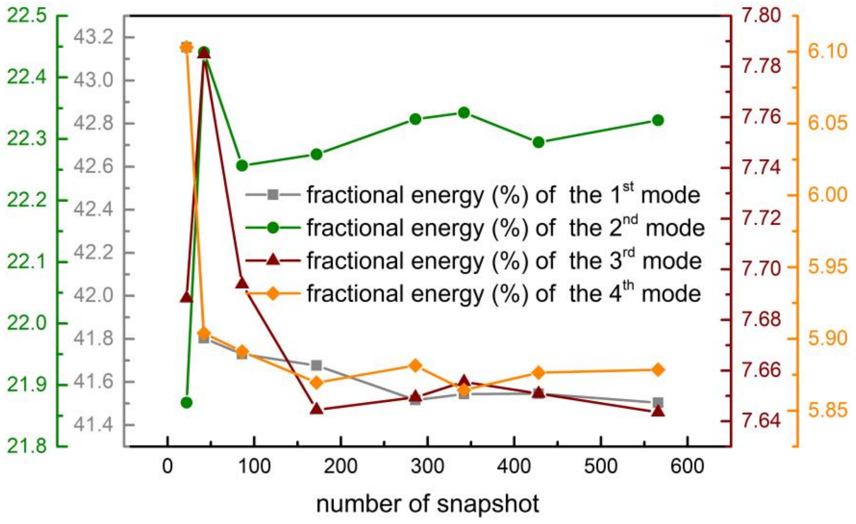

Figure.2. Effect of the number of used modes in POD analysis on fractional energy spectra. In snapshot POD, function of mode number corresponding to different frame rates
